# Formononetin Protects LPS-Induced Mastitis Through Suppressing Inflammation and Enhancing Blood-Milk Barrier Integrity *via* AhR-Induced Src Inactivation

**DOI:** 10.3389/fimmu.2022.814319

**Published:** 2022-02-03

**Authors:** Kaihe Xiang, Peng Shen, Ziyang Gao, Zhuoyu Liu, Xiaoyu Hu, Bin Liu, Yunhe Fu

**Affiliations:** ^1^ Department of Clinical Veterinary Medicine, College of Veterinary Medicine, Jilin University, Changchun, China; ^2^ Department of Clinical Veterinary Medicine, College of Agriculture, Eastern Liaoning University, Dandong, China; ^3^ Cardiovascular Disease Center, First Hospital of Jilin University, Changchun, China

**Keywords:** formononetin, LPS, mastitis, AhR, NF-κB

## Abstract

Formononetin (FOR), a natural flavonoid derived from *Radix Astragali*, has been reported to have anti-inflammatory and anti-oxidative effects. However, its protective mechanism against mastitis is still unknown. Nuclear factor kappa-B (NF-κB) signaling pathway plays an important role in inflammation, especially mastitis. Aryl hydrocarbon receptor (AhR) is involved in inflammatory regulation and defense against diseases. We investigated the protective effect of FOR on LPS-induced mastitis in mice and the effect of Ahr and NF-κB signaling pathways on the development of mastitis. In this study, mastitis model was induced by LPS injection through the nipple duct. Protective effect of FOR on LPS-induced mastitis was assessed by FOR pretreatment. The protective mechanism of FOR against mastitis was further investigated using LPS stimulation on mouse mammary epithelial cells EpH4-Ev. The results showed that LPS-induced mammary histological injury was inhibited by FOR. FOR significantly inhibited LPS-induced MPO activity. FOR administration enhanced the integrity of blood-milk barrier. *In vitro* and *in vivo* experiments showed that FOR inhibited LPS-induced NF-κB signaling pathway activation and the production of inflammatory factors TNF-α and IL-1ß. Moreover, FOR increased the expression of tight junction protein and enhanced blood-milk barrier integrity. LPS activated AhR and Src expression. But FOR induced significant increase in AhR inhibited Src phosphorylation to exert anti-inflammatory effects. In addition, AhR antagonist CH223191 reversed the inhibition of FOR on Src expression. And the inhibition of FOR on NF-κB activation and inflammatory cytokine production were reversed by AhR antagonist CH223191. In conclusion, FOR had protective effects against LPS-induced mastitis *via* suppressing inflammation and enhancing blood-milk barrier integrity *via* AhR-induced Src inactivation.

## Introduction

Mastitis is an inflammatory response of mammary tissues caused by various reasons (1). Among these factors, bacteria have been known as the major cause for mastitis (2). Bacteria can reach the mammary gland and cause the inflammatory response in the mammary tissues (3). Lipopolysaccharide (LPS), a chemical component unique to the outer membrane of Gram-negative bacteria, could trigger an inflammatory response through increasing the production of inflammatory cytokines (4). Elevated inflammatory cytokines were observed in patients of mastitis and LPS-induced mouse mastitis model (5). LPS also induces the production of oxidative mediator and studies showed that both inflammatory response and oxidative stress were participated in the progression of mastitis (6, 7).

Medicinal herbs have been used to treat mastitis for a long time (8). Studies showed that a large body of herbal medicines had anti-inflammatory and anti-oxidative effects (9, 10). Formononetin (FOR), a flavonoid isolated from *Radix Astragali*, has been described to have anti-inflammatory and anti-oxidative activities (11). It has been reported FOR could protect rats against myocardial ischemia/reperfusion injury (12). Previous evidence suggested that FOR had protective effects against LPS-induced acute lung injury in mice (13). FOR also protected mice against concanavalin-A-induced autoimmune hepatitis through inhibiting inflammatory response (14). FOR could inhibit airway inflammation in mouse allergic asthma. *In vitro*, FOR significantly attenuated the expression of LPS-induced inflammatory cytokine in RAW264.7 cells (15). In addition, FOR could inhibit IL-1-induced inflammatory mediator in rat chondrocytes (16). In this study, we detected the effect of FOR on the protection against LPS-induced mastitis in mice. Our findings demonstrated that FOR protected mice against mastitis through inhibiting inflammation and enhancing blood-milk barrier integrity.

## Materials And Methods

### Reagents

FOR (Catalog No. 94334), FITC-albumin (Catalog No. A9771), DAPI (Catalog No. D9542) and LPS (*Escherichia coli* serotype 055: B5, Catalog No. L2880) were obtained from Sigma (St. Louis, MO, USA). The phospho-IκBα (Tyr305, Catalog No. AF3239), NF-κB p65 (Ser311, Catalog No. AF3389), AhR (Ser36, Catalog No. AF3278) and Src (Tyr419, Catalog No. AF3162) antibodies were purchased from Affinity Biosciences (Changzhou, Jiangsu, China). ELISA kits for TNF-α (Catalog No. 430907) and IL-1β (Catalog No. 432601) were acquired from BioLegend (San Diego, CA, USA). EpH4-Evs were obtained from the American Type Culture Collection (ATCC, ATCC^®^ CRL-3063™).

### Animals

Thirty female lactating C57BL/6 mice (7-9 weeks old) were purchased from Liaoning changsheng Biotechnology Co., Ltd. (Benxi, Liaoning, China). The mice were housed in individually ventilated cages and given enough diet and water at 25 ± 1°C. Approval for this experiment was obtained from the institutional animal care and use committee of Jilin University. LPS (10 mg/kg) was dissolved in 50 μL phosphate-buffered saline (PBS) and injected into the breast ducts of L4 (on the left) and R4 (on the right) for 24 h. FOR (10, 20, 30 mg/kg) were dissolved in normal saline and administered 1 h prior LPS treatment.

### Histological Analysis

The collected mammary glands were fixed in 4% paraformaldehyde. Hematoxylin and eosin (H&E) staining was used, followed by observation of morphological changes in breast tissues under a microscope and digital camera (Olympus, Tokyo, Japan).

### MPO Assay

24 h after LPS treatment, the mammary glands were collected and the myeloperoxidase (MPO) level in mammary tissues was determined using the detection kit (Catalog No. A044-1-1, Nanjing Jiancheng Bioengineering Institute, Nanjing, China) according to the manufacturer’s guidelines.

### ELISA Assay

The mammary glands were collected in the same way as before. ELISA kits (BioLegend, CA, USA) were utilized to detect the production of TNF-α and IL-1ß in the mammary tissues.

### Western Blot Analysis

The protein extraction kit (Catalog No. P0013M, Beyotime Biotechnology, Shanghai, China) was used for the extraction of total protein from cells. The concentration of protein was measured with the BCA kit (Catalog No. P0012S Beyotime Biotechnology, Shanghai, China). 30 μg protein was loaded by 12% SDS-PAGE and transferred to the PVDF membranes. Then, the membranes were blocked with 5% BSA and incubated with NF-κB and AhR signaling pathway antibodies. Finally, the membranes were incubated with secondary antibodies and detected using the ECL chemiluminescence reagent.

### Immunofluorescence

The collected mammary glands were fixed in 4% paraformaldehyde, embedded in paraffin and tissue sections were made. The sections were then dewaxed and antigenically repaired. 5% goat serum was used to block nonspecific interactions, overnight at 4°C. 50 mg/mL FITC-albumin mother solution was prepared with double-distilled water and diluted 40-fold with 5% goat serum. The sections were washed with PBS and incubated with FITC-albumin at 4°C overnight. The sections were washed with PBS containing 0.1% Triton and stained with DAPI to avoid light for 5-8 minutes. Finally, the sections were sealed with glycerin and observed as soon as possible.

### *In Vitro* Experiment

EpH4-Ev cells were incubated in DMEM supplemented with 10% fetal bovine serum (FBS) at 37°C. The effect of FOR on cell viability was measured by MTT assay. Then, the cells were pre-treated with FOR (10, 20, and 30 μM) 1 h before LPS treatment. The cytokines were measured by ELISA 24 h after LPS treatment (1μg/mL). The key protein levels involved in the NF-κB signaling pathway were detected 30 min after LPS treatment by western blot. For AhR inhibitory experiment, cells were pretreated with FOR (30 μM) and CH223191 (10 μM) for 1 h, and then stimulated with LPS for indicated times.

### Statistical Analyses

All the results were expressed as means ± SEM. The data were analyzed using one way analysis of variance (ANOVA) followed by the Tukey’s *post hoc* test. Value of *P* < 0.05 was considered to be significant.

## Results

### FOR Attenuates LPS-Induced Mammary Histopathological Alterations

**Figure 1:**
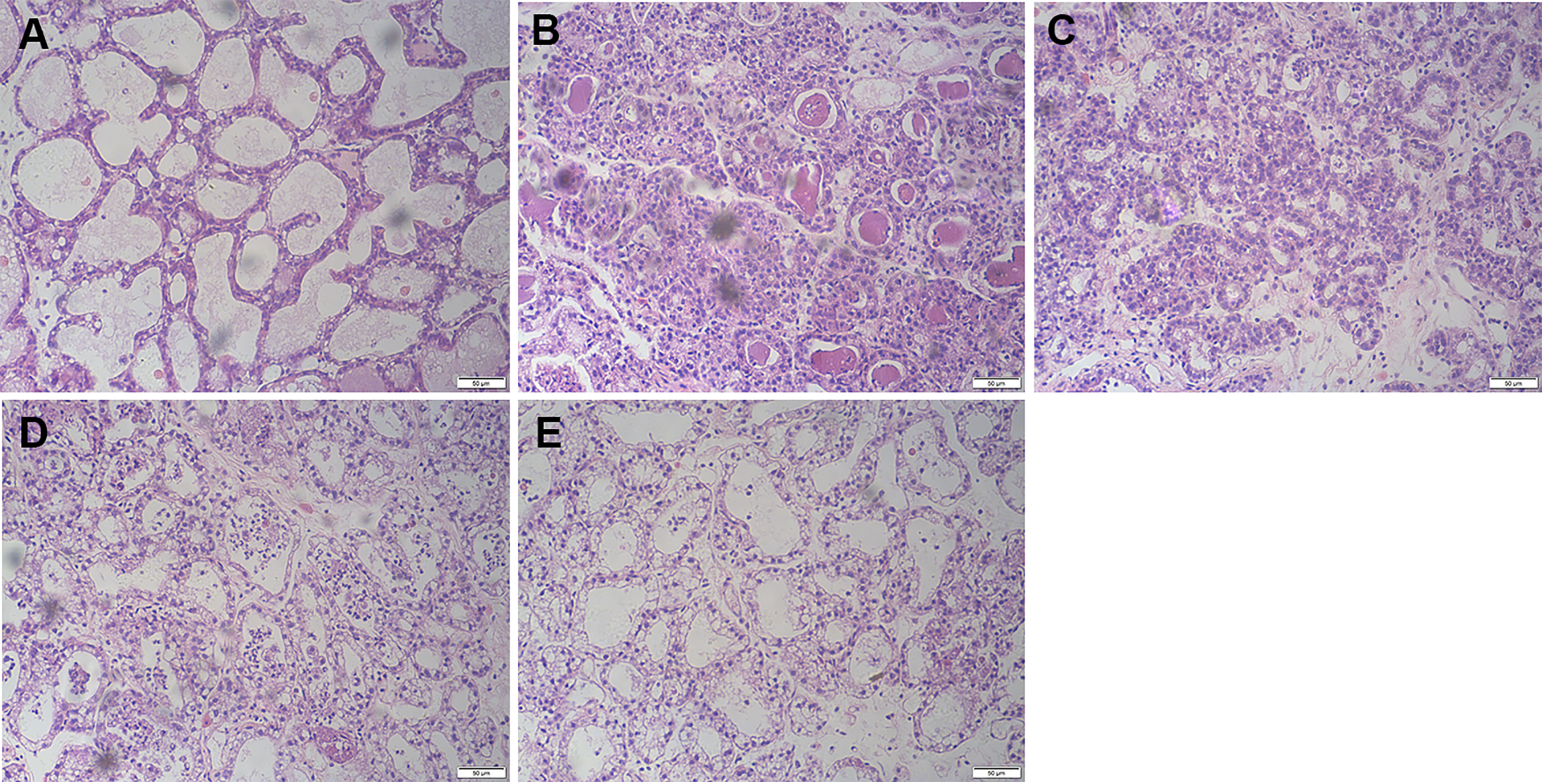
Effects of FOR on LPS-induced histopathological changes in mice with mammary (magnification 200×). **(A)** Control group: normal mammary gland tissue structure. **(B)** LPS group: severe inflammatory cell infiltration. **(C–E)** LPS + FOR (10, 20, 30 mg/kg) groups: gradually decreasing inflammatory cell infiltration after administration of FOR.

After LPS treatment, typical inflammatory manifestations such as redness, swelling, heat, and pain began to appear in the mammary tissue. Compared to normal breast tissue (**Figure 1A**), H&E staining showed serious mammary histopathological changes, such as inflammatory cell infiltration, edema, and hyperemia (**Figure 1B**). It suggested that LPS was capable of inducing mastitis in mice. However, LPS-induced mammary histopathological changes were inhibited by FOR. The histopathological changes in the mammary glands of mice gradually diminished as the increasing concentration of FOR (**Figures 1C** **–E**).

### FOR Suppresses LPS-Induced MPO Activity

**Figure 2:**
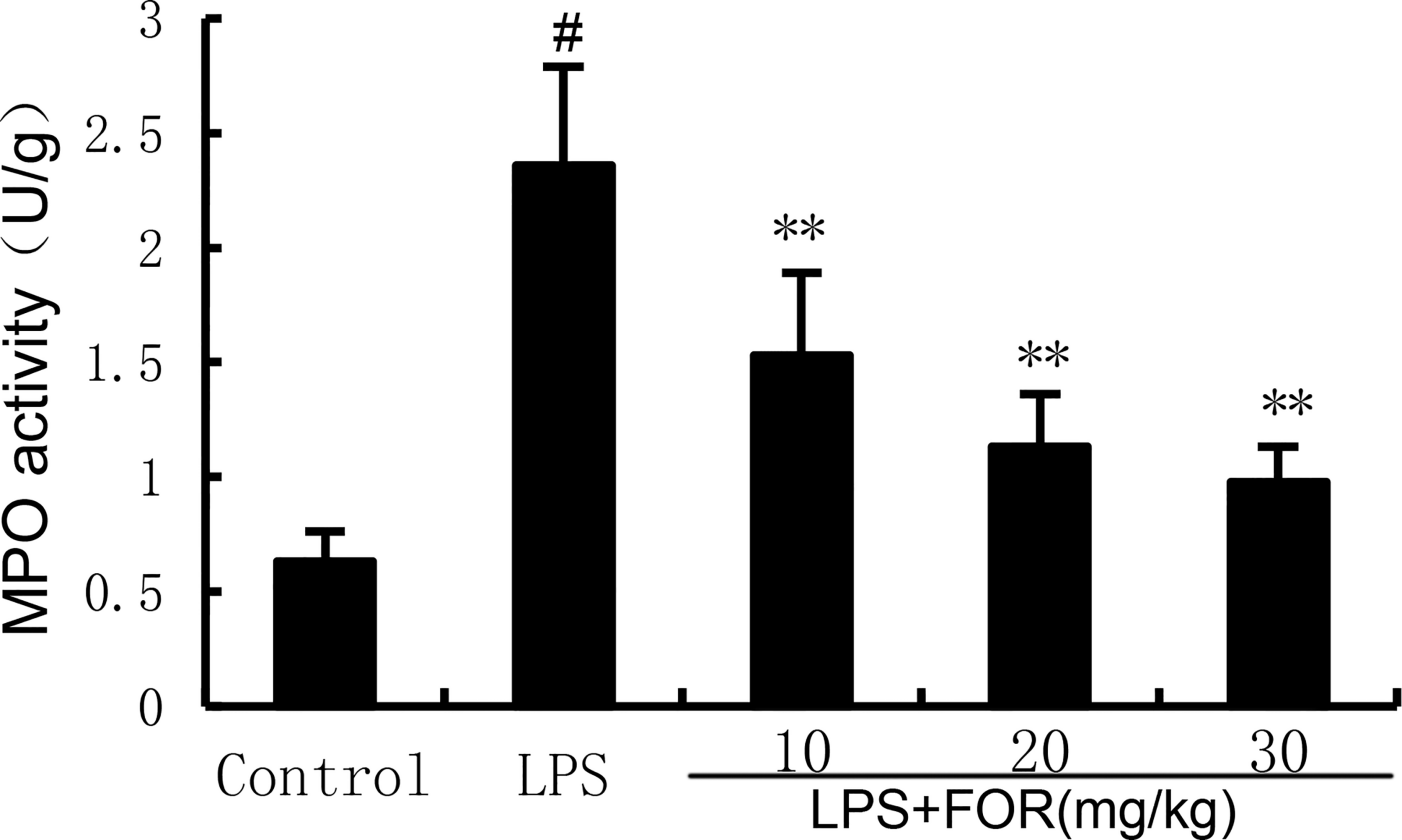
FOR suppresses LPS-induced MPO activity. The results are expressed as the mean ± SEM of triple parallel measurements. ^#^(*P* < 0.01) is significantly different from control group; **(*P* < 0.01) is significantly different from LPS group.

The MPO activity increased significantly in the LPS group in comparison to the controls. Conversely, the MPO activity in the LPS+FOR group was much lower when compared to the LPS group (**Figure 2**). This result suggested FOR could inhibit the infiltration of neutrophils into lung tissues.

### FOR Suppresses TNF-α and IL-1β Production Induced by LPS

**Figure 3:**
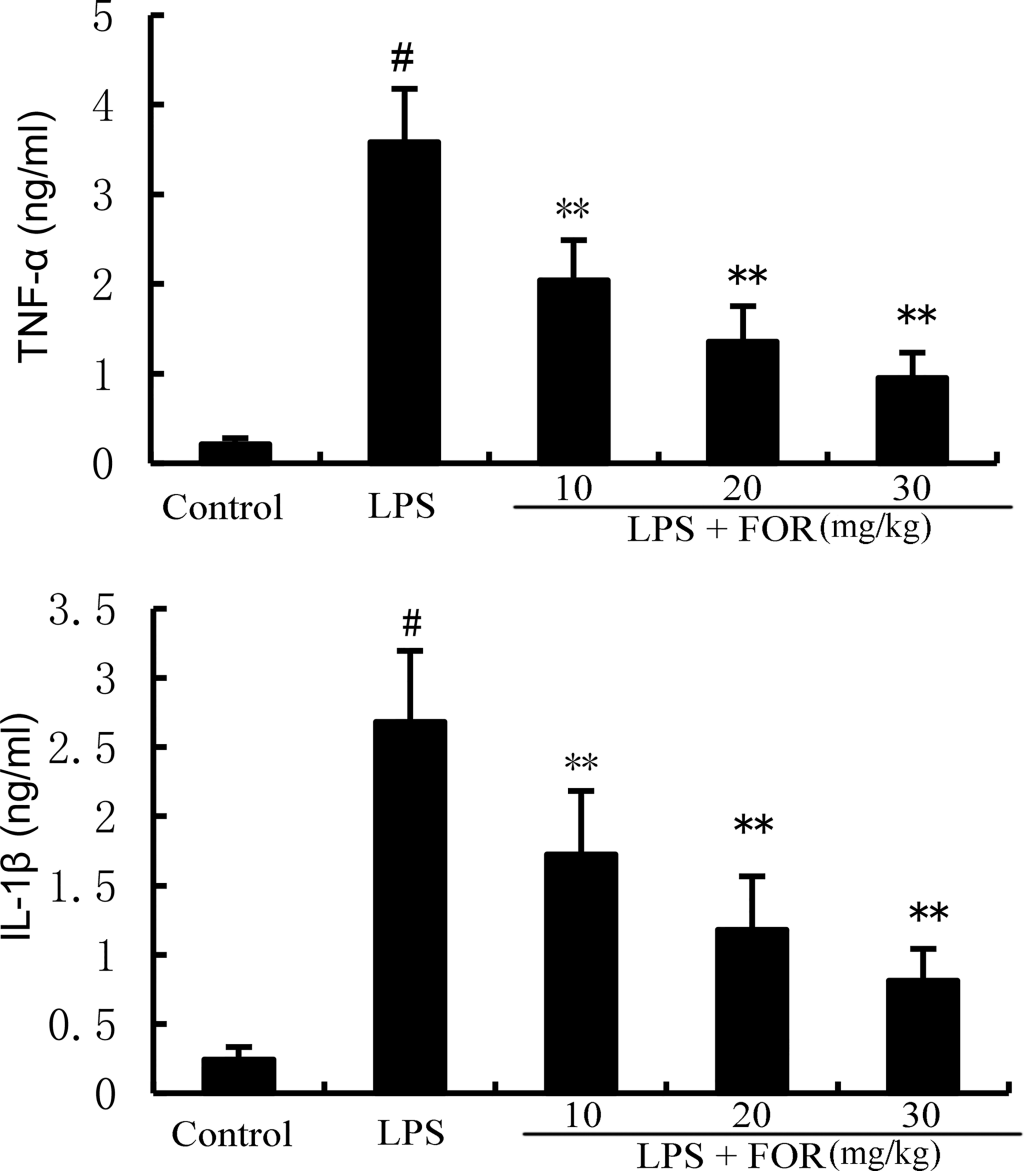
FOR decreases LPS-induced generation of TNF-α and IL-1β in breast tissue. The data herein are expressed as mean ± SEM obtained from triple parallel measurements. ^#^(*P* < 0.01) is significantly different from control group; **(*P* < 0.01) is significantly different from LPS group.

**Figure 4:**
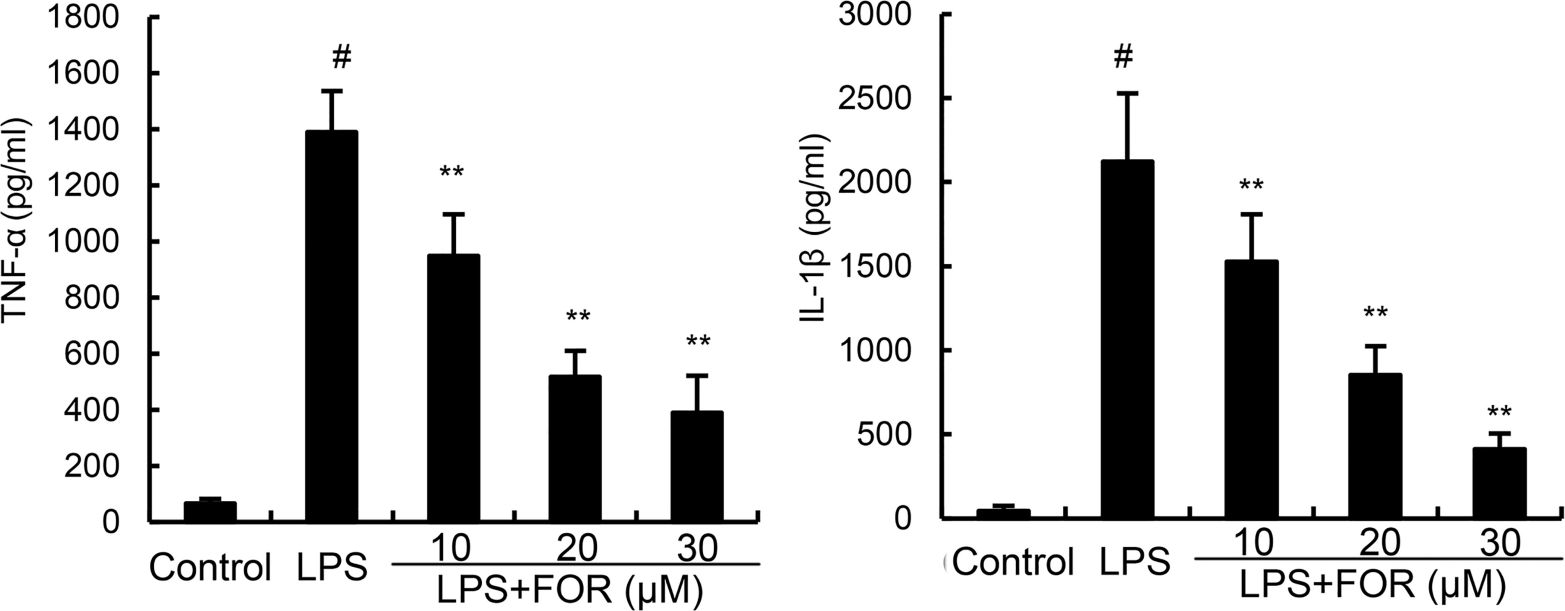
FOR reduces LPS-induced secretion of TNF-α and IL-1β in EpH4-Ev cells. The results herein are expressed as mean ± SEM of triple parallel measurements. ^#^(*P* < 0.01) is significantly different from control group; **(*P* < 0.01) is significantly different from LPS group.

TNF-α, and IL-1β levels in mammary tissues were significantly elevated as compared to the control group in the LPS group. In contrast, TNF-α and IL-1β levels of mammary tissues were much lower in the LPS+FOR group comparing to the LPS group (**Figure 3**). *In vitro*, treatment of FOR (10, 20, and 30 μM) also inhibited LPS-induced TNF-α and IL-1β production in EpH4-Ev cells (**Figure 4**). These results suggested FOR could inhibit LPS-induced inflammatory response.

### Effects of FOR on Tight Junction Expression

**Figure 5:**
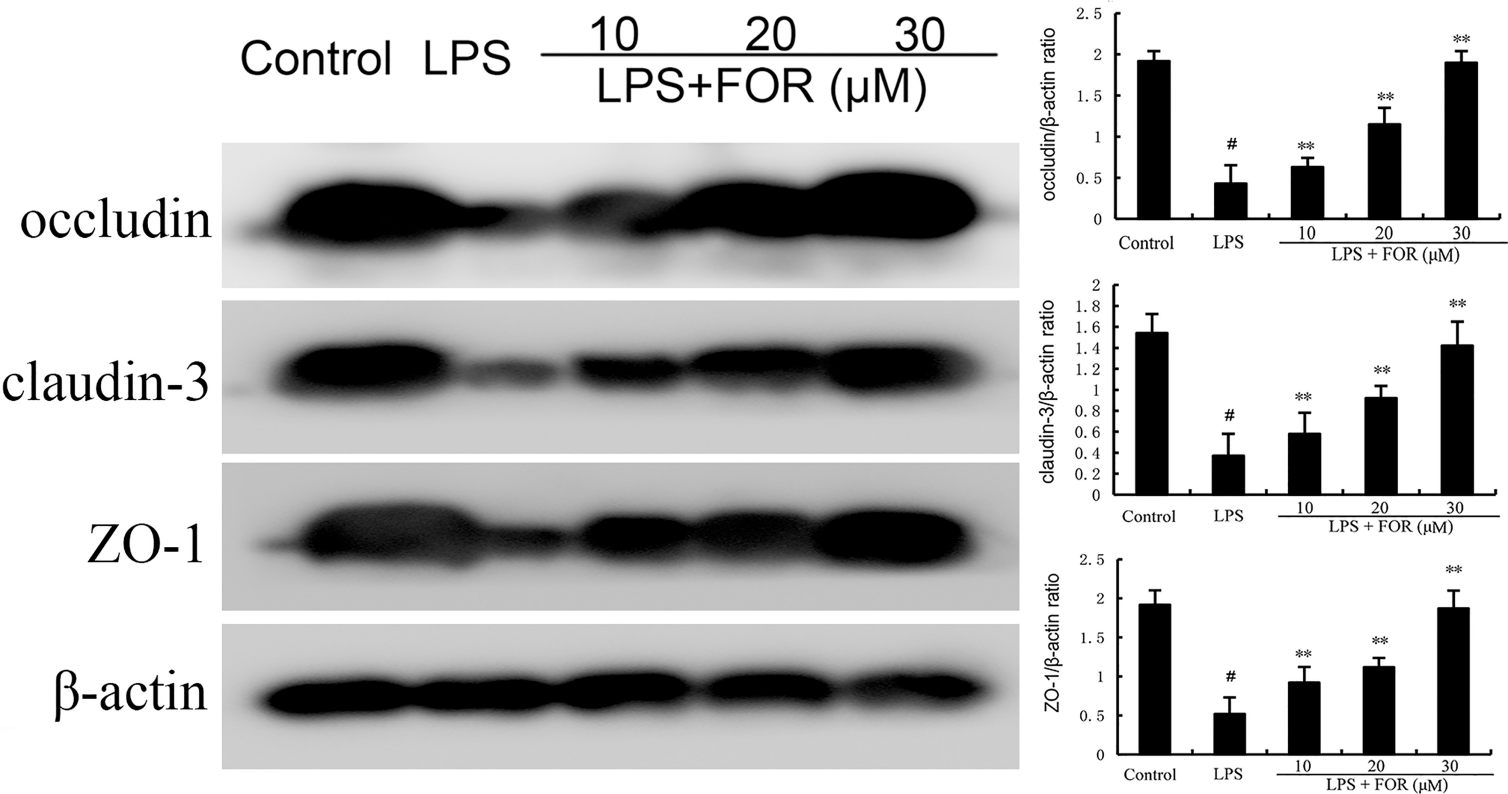
FOR promotes the expression of tight junction-related proteins. The present results are displayed as the mean ± SEM of triple parallel measurements. ^#^(*P* < 0.01) is significantly different from control group; **(*P* < 0.01) is significantly different from LPS group.

The effects of FOR on blood-milk barrier integrity were assessed by detecting the expression of tight junction protein claudin-3, occludin, and ZO-1. The results showed that claudin-3, occludin, and ZO-1 expression decreased significantly in the LPS group in comparison with controls. In contrast, claudin-3, occludin, and ZO-1 expression were increased by FOR compared to LPS group (**Figure 5**).

### FOR Suppresses the LPS-Induced Blood-Milk Barrier Disruption

**Figure 6:**
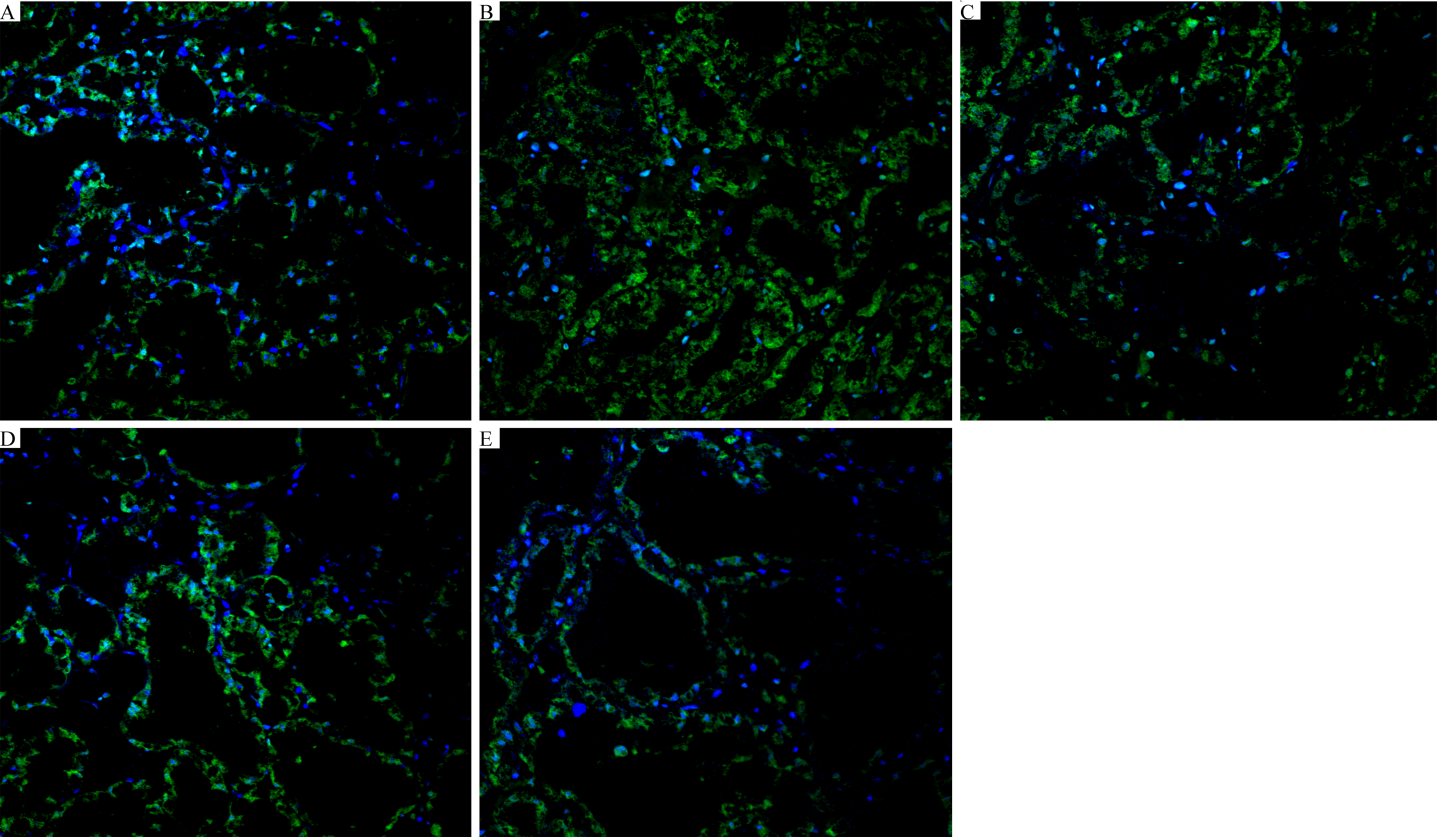
FOR suppresses the LPS-induced blood-milk barrier disruption (magnification 400×). **(A)** Control group: normal mammary gland tissue structure. **(B)** LPS group: severe FITC-albumin infiltration and exfoliation of epithelial cells. **(C–E)** LPS + FOR (10, 20, 30 mg/kg) groups: gradually intact blood-milk barrier after administration of FOR. Blue spots represented the nuclei; green spots were albumin.

The localization of FITC-albumin was observed by immunofluorescence detection. As shown in **Figure 6**, green and blue show FITC-albumin and nuclei (DAPI), respectively. The infiltration of large amounts of FITC-albumin into the lumen of the glandular vesicles indicated that LPS was capable of causing blood-milk barrier disruption (**Figure 6B**). However, FOR significantly inhibited LPS-induced increase of blood-milk barrier permeability (**Figures 6C** **–E**).

### FOR Suppresses the LPS-Induced NF-κB Activation

**Figure 7:**
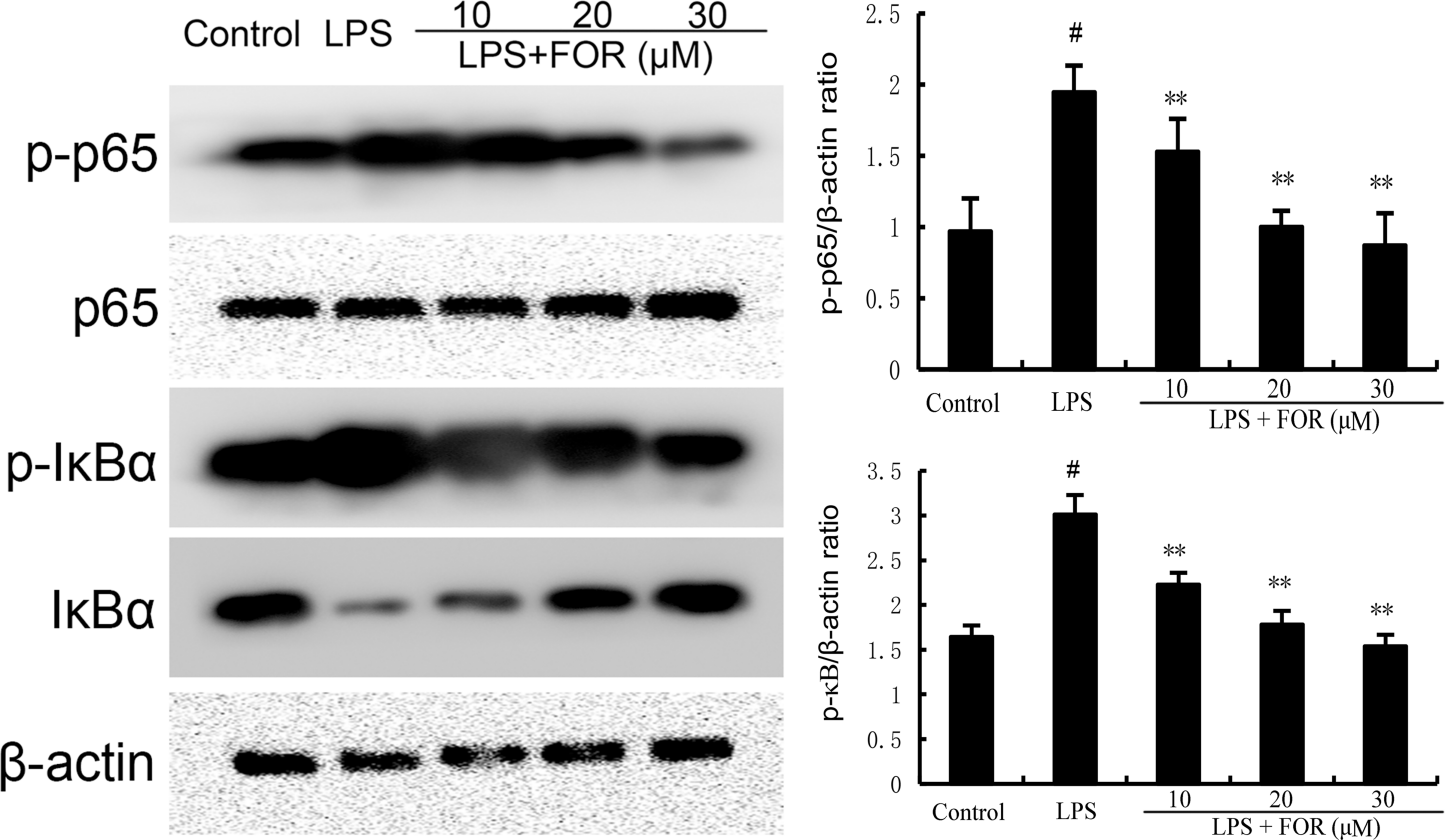
FOR inhibits LPS-induced NF-κB activation. The present results are displayed as the mean ± SEM of triple parallel measurements. ^#^(*P* < 0.01) is significantly different from control group; **(*P* < 0.01) is significantly different from LPS group.

NF-κB is a crucial transcription factor that regulates the production of inflammatory mediators. As the results shown in **Figure 7**, the phosphorylation levels of NF-κB and IκBα increased significantly in the LPS group in comparison to the controls. Conversely, the phosphorylation levels of NF-κB and IκBα in the LPS+FOR groups were much lower when compared to the LPS group (**Figure 7**). These findings suggested FOR suppressed LPS-induced inflammatory response *via* inhibition of the NF-κB signal pathway.

### Effects of FOR on AhR and Src Expression

**Figure 8:**
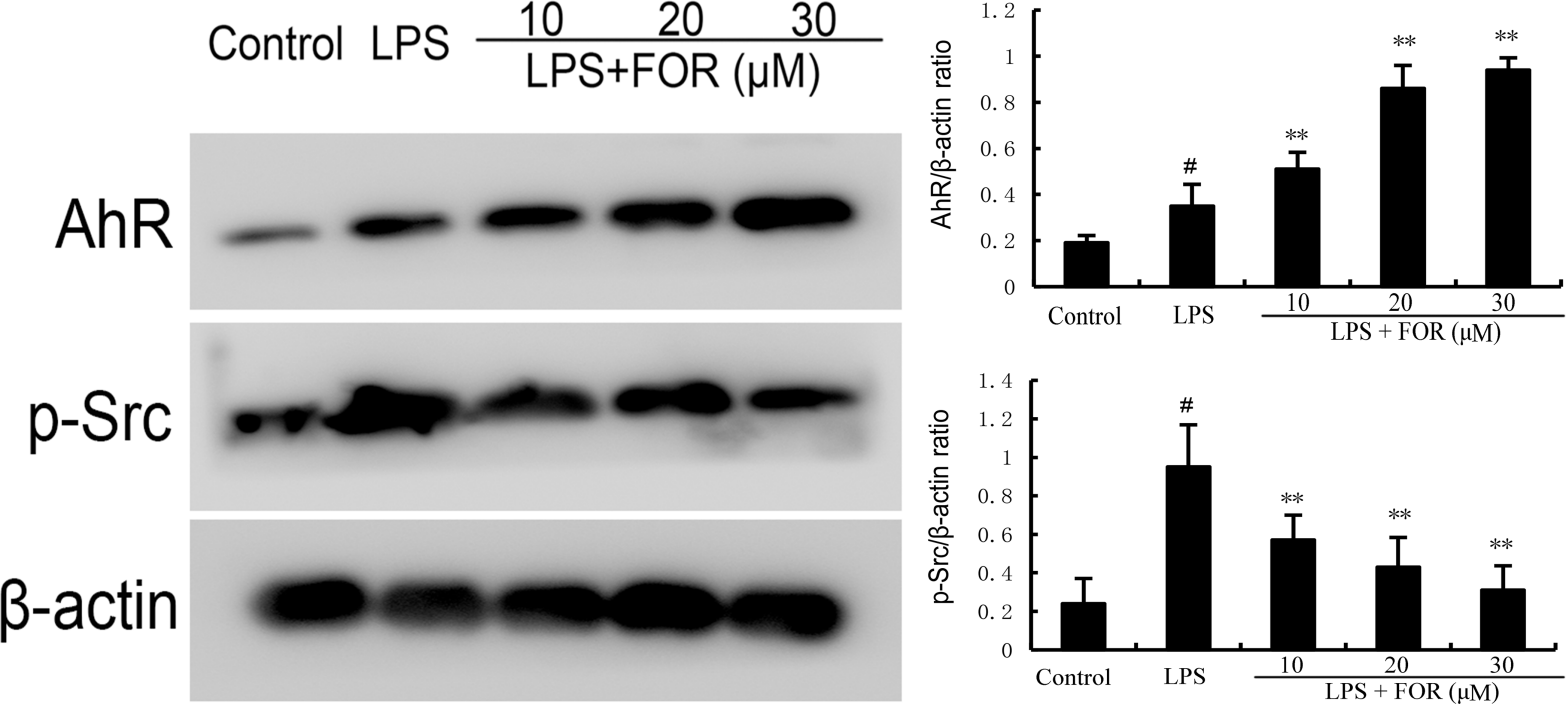
FOR increases AhR expression and inhibits the phosphorylation level of Src. The present results are displayed as the mean ± SEM of triple parallel measurements. ^#^(*P* < 0.01) is significantly different from control group; **(*P* < 0.01) is significantly different from LPS group.

AhR/Src signaling pathway participated in the regulation of inflammation. As shown in **Figure 8**, FOR increased the expression of AhR in a concentration-dependent manner. Meanwhile, LPS increased the phosphorylation level of Src and the increase was inhibited by the treatment of FOR (**Figure 8**).

### FOR Inhibits LPS-Induced Inflammation *via* AhR-Induced Src Inactivation

**Figure 9:**
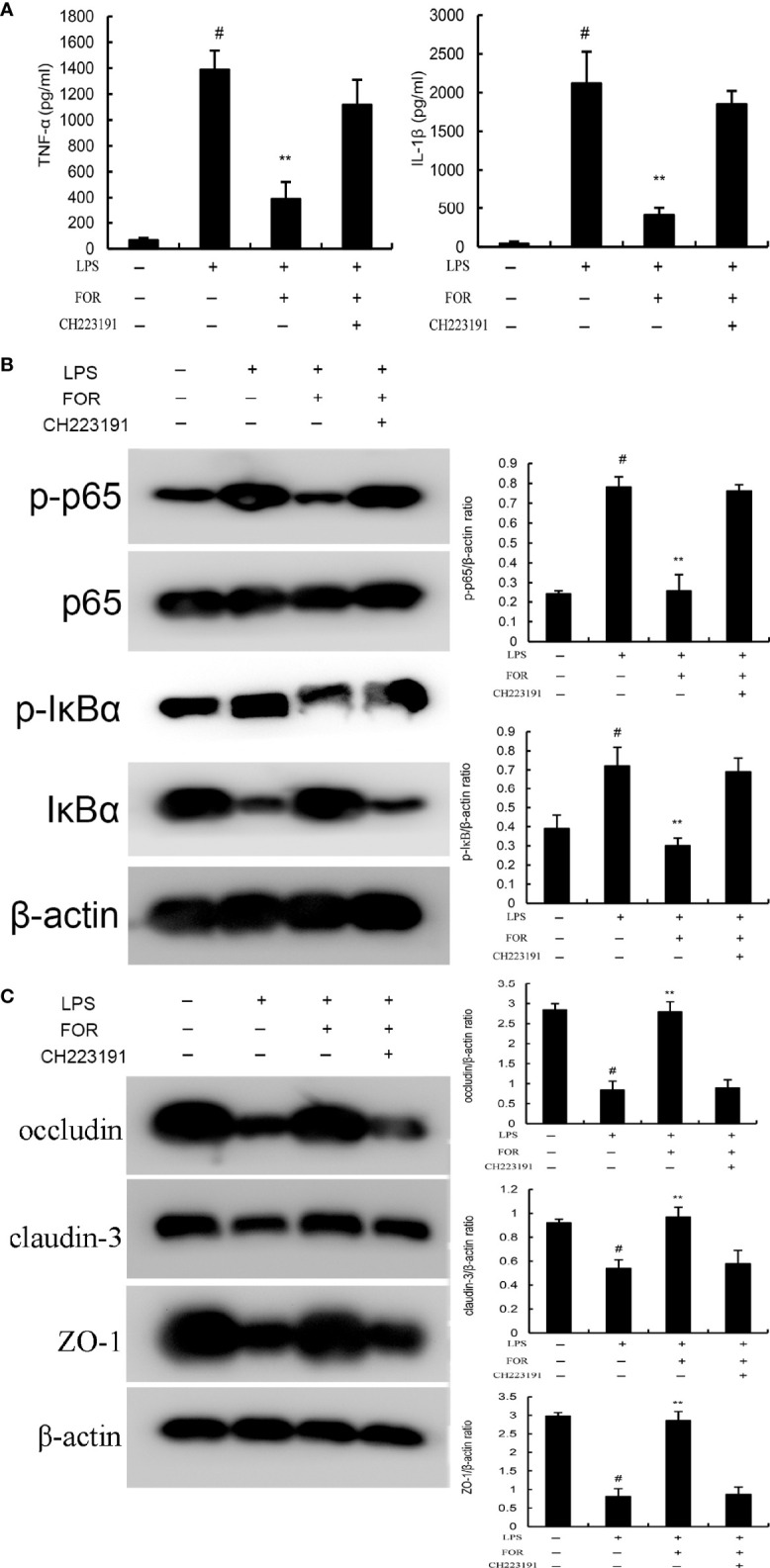
FOR inhibits LPS-induced inflammation *via* AhR-induced Src inactivation. **(A)** The levels of proinflammatory cytokines TNF-α and IL-1β in EpH4-Evs cells. **(B) **The expression of NF-κB signaling pathway. **(C) **The expression of tight junction proteins occludin, claudin-3, and ZO-1 in EpH4-Evs cells. The present results are displayed as the mean ± SEM of triple parallel measurements. ^#^(P < 0.01) is significantly different from control group; **(P < 0.01) is significantly different from LPS group.

LPS was used to stimulate EpH4-Evs cells, and cell viability was measured by MTT assay. When there was a significant decrease in cell viability and a significant increase in inflammatory factors, it suggested that LPS had a significant inflammatory effect on EpH4-Evs cells. The optimal concentration and time of LPS stimulation on EpH4-Evs cells were determined by pre-experiments. To further clarify the anti-inflammatory mechanism of FOR, AhR antagonist CH223191 was used in this study. The results showed that the inhibitory effects of FOR to NF-κB activation (**Figure 9B**) and inflammatory cytokine generation (**Figure 9A**) were reversed by AhR antagonist CH223191. Furthermore, the enhanced blood-milk barrier integrity induced by FOR was also inhibited by AhR antagonist CH223191 (**Figure 9C**). These results indicated FOR inhibited LPS-induced inflammation and enhanced blood-milk barrier integrity *via* AhR-induced Src inactivation.

## Discussion

In this study, we evaluated the protection of FOR against LPS-induced mastitis. The principal findings are as follows FOR significantly inhibited infiltration of neutrophils, inflammatory cytokine production, and MPO activity in LPS-induced mastitis in mice. Its mechanism was by suppressing inflammation and enhancing blood-milk barrier integrity.

Inflammation was involved in the development of mastitis (17). The invading bacteria could activate the innate immune system and lead to the production of inflammatory and oxidative mediators (18). These increased inflammatory mediators such as TNF-α, IL-1ß and IL-6 contributed to the pathogenesis of mastitis (19). They could cause damage to breast tissue and cells (20). In the present study, it was demonstrated that FOR remarkably suppressed the generation of LPS-induced inflammatory cytokine. In combined with the mammary histopathological results, the results suggested FOR suppressed LPS-induced mastitis through attenuating inflammatory response.

The integrity of blood-milk barrier is the premise to ensure the normal lactation function of mammary tissue (21). It has been known that tight junction is the key structure to maintain the integrity of blood-milk barrier (22). It was found that the integrity of blood-milk barrier was destroyed when mastitis occurred (23). Blood-milk barrier dysfunction could lead to excessive neutrophils entering the mammary tissue and causing damage (24). Therefore, maintaining the integrity of blood-milk barrier is of great significance to treat mastitis. In this study, our results showed that FOR could increase the expression of tight junction proteins in the mammary epithelium and enhance the integrity of the blood-milk barrier.

NF-κB is an important molecule that regulates the transcription of inflammatory cytokines (25). It was involved in the regulation of inflammatory cytokine production and employed as a target for the treatment of many inflammatory diseases (26). In recent years, many natural herbal medicines have been found to protect mice against mastitis through inhibiting NF-κB activation (27, 28). And we found that FOR was able to significantly inhibit LPS-induced NF-κB activation. AhR is a ligand activated transcription factor (29). Previous research has demonstrated the role of AhR in the metabolism of poisons and chemicals in the environment, regulating biological rhythm, reproduction and oxidative stress (30). Recently, it has been found that AhR has important functions in innate and adaptive immune response, autoimmune diseases, infection and inflammation (31, 32). AHR may function as a pattern recognition receptor in host resistance to pathogenic microorganisms (32). LPS can activate AHR, which decreases the expression of pro-inflammatory cytokines and regulates long-term systemic inflammation (33). In addition, AHR is a component of a retinoic acid-induced signalsome involved in neutrophilic granulocyte differentiation (34). AHR and NF-κB signaling pathways interact with each other. LPS-induced activation of NF-κB in the mouse thymus involves RelA-dependent AhR expression and enhanced activity of AhR-regulated genes (35). Src is a nonreceptor protein tyrosine kinase that is capable of forming a complex with AhR (36). AhR deficiency results in enhanced phosphorylation of Src (37). Recent studies demonstrated that activation of AhR could lead to the inhibition of Src, thus increasing the expression of anti-inflammatory cytokines and enhancing intestinal barrier function to alleviate inflammation (36, 38). In the present work, it was shown that LPS activated AhR and Src expression and correlated closely with NF-κB signaling pathway activation. However, FOR could concentration-dependently increase the expression of AhR and inhibit Src phosphorylation to exert anti-inflammatory effects. Furthermore, AhR antagonist reversed the inhibitory effects of FOR on NF-κB activation and inflammatory factor production. The enhanced blood-milk barrier integrity induced by FOR was also inhibited by AhR antagonist.

## Conclusions

In conclusion, the results indicated the protective effect of FOR on LPS-induced mastitis through suppressing inflammation and enhancing blood-milk barrier integrity. FOR can be a potential therapeutic agent for mastitis.

## Data Availability Statement

The original contributions presented in the study are included in the article/supplementary material. Further inquiries can be directed to the corresponding authors.

## Ethics Statement 

The animal study was reviewed and approved by Institutional Animal Care and Use Committee of Jilin University.

## Author Contributions

YF and BL designed the experiment. KX, PS, ZG, and ZL did the experiment. XH and PS revised the manuscript. YF analyzed the data. KX and BL wrote the paper. All authors contributed to the article and approved the submitted version.

## Funding

The work was supported by the National Natural Science Foundation of China (Nos. 32122087).

## Conflict of Interest

The authors declare that the research was conducted in the absence of any commercial or financial relationships that could be construed as a potential conflict of interest.

## Publisher’s Note

All claims expressed in this article are solely those of the authors and do not necessarily represent those of their affiliated organizations, or those of the publisher, the editors and the reviewers. Any product that may be evaluated in this article, or claim that may be made by its manufacturer, is not guaranteed or endorsed by the publisher.
